# Spatial Stream Segregation by Cats

**DOI:** 10.1007/s10162-016-0561-0

**Published:** 2016-03-18

**Authors:** Lauren K. Javier, Elizabeth A. McGuire, John C. Middlebrooks

**Affiliations:** Department of Neurobiology & Behavior, University of California at Irvine, Irvine, CA USA; Center for Hearing Research, University of California at Irvine, Irvine, CA USA; Department of Otolaryngology, University of California at Irvine, Irvine, CA 92697-5310 USA; Department of Cognitive Sciences, University of California at Irvine, Irvine, CA USA; Department of Biomedical Engineering, University of California at Irvine, Irvine, CA USA

**Keywords:** auditory scene analysis, spatial hearing, binaural hearing, auditory spatial cues, release from masking, cocktail party effect

## Abstract

Listeners can perceive interleaved sequences of sounds from two or more sources as segregated streams. In humans, physical separation of sound sources is a major factor enabling such stream segregation. Here, we examine spatial stream segregation with a psychophysical measure in domestic cats. Cats depressed a pedal to initiate a target sequence of brief sound bursts in a particular rhythm and then released the pedal when the rhythm changed. The target bursts were interleaved with a competing sequence of bursts that could differ in source location but otherwise were identical to the target bursts. This task was possible only when the sources were heard as segregated streams. When the sound bursts had broad spectra, cats could detect the rhythm change when target and competing sources were separated by as little as 9.4°. Essentially equal levels of performance were observed when frequencies were restricted to a high, 4-to-25-kHz, band in which the principal spatial cues presumably were related to sound levels. When the stimulus band was restricted from 0.4 to 1.6 kHz, leaving interaural time differences as the principal spatial cue, performance was severely degraded. The frequency sensitivity of cats in this task contrasts with that of humans, who show better spatial stream segregation with low- than with high-frequency sounds. Possible explanations for the species difference includes the smaller interaural delays available to cats due to smaller sizes of their heads and the potentially greater sound-level cues available due to the cat’s frontally directed pinnae and higher audible frequency range.

## **INTRODUCTION**

In typical auditory environments, listeners show a remarkable ability to isolate sounds of interest amid other competing sounds. This has been referred to as the cocktail party effect (after Cherry 1953) or auditory scene analysis (Bregman 1990). One key element of auditory scene analysis is stream segregation, which permits listeners to disentangle multiple temporally interleaved sequences of sounds. An example of stream segregation is that of a listener streaming together sequences of syllables as sentences from one talker while rejecting syllables from one or more other competing talkers. Multiple acoustic features enable stream segregation, including fundamental frequency, temporal envelope, bandwidth, phase, and lateralization (Moore and Gockel 2002). The present study focuses on the contribution of spatial separation between the sources of the target and distracting sounds.

Previous research in our laboratory has evaluated spatial stream segregation by human listeners, using a non-verbal, objective measure. Listeners were asked to discriminate rhythms of target sequences of broadband noise bursts that were masked by interleaved noise-burst sequences (Middlebrooks and Onsan 2012). Performance was at chance levels when the target and masker sources were co-located, but improved with increasing target/masker spatial separation. The median rhythmic masking release threshold was 8.1°, which approached those listeners’ minimum audible angles for discriminating changes in source locations of single sound bursts. Performance was not significantly different when the noise bursts were band-limited from 0.4 to 1.6 kHz, but thresholds broadened significantly to a median of 15.9° when tested with bursts band-limited from 4 to 16 kHz. Those results suggest that interaural time differences (ITD) in temporal fine structure were the acoustic cues that provided the highest spatial acuity for humans in that task. A related study examined correlates of spatial stream segregation by neurons in cortical area A1 of anesthetized cats (Middlebrooks and Bremen 2013). Neurons synchronized preferentially to one or the other of two interleaved sound sequences from spatially separated sources with spatial acuity approaching that of the human listeners in the psychophysical task. Contrary to the expectation from the human results, however, acuity of spatial stream segregation by cat neurons was by most tests finer among neurons tuned to high frequencies than among those tuned to low frequencies.

The purpose of the present study was to evaluate the spatial acuity of stream segregation in cats, thereby providing psychophysical data for the same species in which data from single cortical neurons can be obtained. In particular, we wished to test whether the cat listeners showed finer spatial acuity at low frequencies, like the human listeners, or finer acuity at high frequencies, consistent with the cat cortical physiology. For this study, we modified the task from the two-alternative task employed in the previous human psychophysical study (Middlebrooks and Onsan 2012) to a hold-release paradigm. Cats depressed a pedal to begin presentation of a standard sound sequence, Rhythm 1, and then released the pedal when the sequence changed to Rhythm 2. The target sounds were interleaved with masker sequences that varied in source location from trial to trial. It was necessary for the cat to segregate target from masker streams in order to detect the change in rhythm and thereby receive a food reward.

The results demonstrate that cats can segregate streams of broadband sounds with spatial acuity nearly as fine as that of humans. Performance was consistently better for high than for low frequencies, consistent with the previous cat physiological results but contrary to the human psychophysics. Factors contributing to that inter-species difference in frequency dependence could include the narrower interaural time differences provided by the smaller head of the cat as well as potentially greater sound-level cues available due to the cat’s frontally directed pinnae and higher audible frequency range.

## **MATERIALS AND METHODS**

### Animals

All procedures were in accordance with the NIH Animal Welfare Guidelines and with a protocol approved by the Institutional Animal Care and Use Committee at the University of California at Irvine. Six male domestic shorthaired cats (*Felis catus*) were obtained from a breeding colony at the University of California at Davis. No hearing deficits were evident. Ages ranged from 2 to 6 months at the beginning of training and from 8 to 36 months at the time of collection of the reported data. Male cats were used exclusively for this study to facilitate group housing. The cats were neutered to reduce aggressive behavior, making it possible to introduce new animals to the colony. Food was restricted on days that animals were performing the behavioral task (5 days a week). On those days, cats received moist food as behavioral reinforcement during training or testing sessions and then were given free access to dry food for up to an hour after the session. On weekends, cats were given free access to dry food for 3 h per day. Water was freely available in the housing area.

### Experimental Apparatus

Experiments were conducted in a double-walled sound-attenuating anechoic chamber (Industrial Acoustics, inside dimensions 2.6 × 2.6 × 2.5 m) lined with SONEXone absorbent foam to suppress sound reflections. The chamber contained 13 8.4-cm-diameter two-way loudspeakers positioned on a horizontal circular hoop, 1.2 m in radius, at azimuths of 0 and ±5, 10, 20, 40, 60, 80° relative to the front of the apparatus. The cat was supported on a raised platform, which was adjusted in height so that the cat’s head was centered in the array of loud speakers. A harness restrained the animal to the platform but permitted free movement of the limbs and head. A feeder was mounted on a pneumatic cylinder located on the animal pedestal. The feeder was raised to provide behavioral reinforcement and was lowered during sound presentation. All behavioral sessions were conducted in the dark and were monitored with video using infrared illumination.

### Stimulus Generation

Stimulus generation and data acquisition used System III hardware from Tucker-Davis Technologies (TDT;Alachua, FL) controlled by custom MATLAB software (The Mathworks; Natick, MA) on a Windows-based computer. Sounds were generated with a 24-bit precision at a sample rate of 97,656 s^−1^. Loudspeakers were calibrated using a precision ½” microphone (ACO Pacific) that was positioned at the center of the apparatus at the normal location of the animal’s head. Golay codes were used as probe sounds (Zhou et al. 1992). The calibration procedure yielded a 1029-tap finite-impulse-response correction filter for each speaker. The filters flattened and equalized the broadband frequency responses of the loudspeakers such that, for each loudspeaker, the standard deviation of the magnitude spectrum across the 0.2–25-kHz calibrated passband was <1 dB. The responses were rolled off by 10 dB from 25 kHz to 40 kHz.

**FIG. 1:**
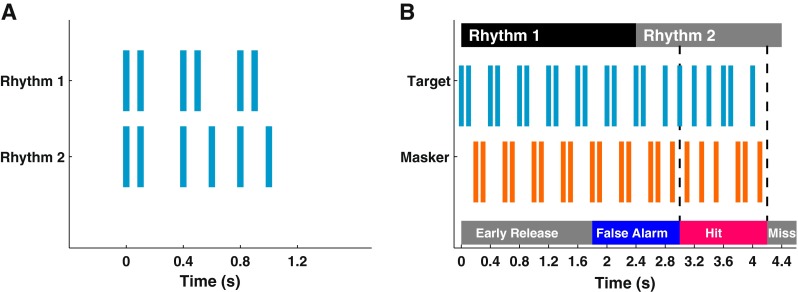
**A** Two temporal patterns of noise bursts, Rhythm 1 and Rhythm 2. **B** Timing of response windows. This example shows the Hold 2 condition. The target and masker sequences were interleaved in time. The masker pattern was complementary to that of the target. The earliest detectable change from Rhythm 1 to Rhythm 2 occurred 600 ms after the onset of Rhythm 2. That time marked the beginning of a 1200-ms time window in which the cat could release the pedal to score a “hit” and receive a food reward. Releases within 1200 ms prior to the beginning of the hit window were scored as “false alarms.” Releases even earlier were scored as “early releases.” Releases after the hit window were scored as “misses”.

Stimuli consisted of sequences of noise bursts generated in real time by gating a continuous Gaussian noise source generated by the TDT RZ6 digital signal processor. Each noise burst was 20 ms in duration, gated with raised cosine functions with 1-ms rise and fall times. The noise presented from each speaker was filtered with the corresponding speaker correction filter and then was band-pass filtered with fourth-order Butterworth filters to one of the following bands: 0.4–25 kHz for the broadband condition, 0.4–1.6 kHz for the low-band condition, and 4–25 kHz for the high-band condition. The filter bands were identical between target and masker sounds and throughout each training or testing session. Sounds were presented at 60 dB SPL for all filter conditions. Target sound sequences were presented with two temporal patterns, referred to as Rhythms 1 and 2 (Fig. 1A). Each trial began with one to four continuous sequences of Rhythm 1, with the number of sequences varying randomly from trial to trial. The Rhythm 1 sequence was followed without interruption by a 1200-ms Rhythm-2 sequence repeated 1.5 times. Masker sound sequences were interleaved with target sequences (Fig. 1B). The masker sequences were exactly complementary to the target sequences such that, when target and masker sources were co-located at 0°, the stimulus was a continuous sequence of undifferentiated noise bursts. The aggregate rates of target and masker noises bursts were 10 s^−1^, meaning that onsets of target or masker bursts were presented at intervals of 100 ms.

### Behavioral Task and Training

The behavioral task was patterned after the hold-release paradigm described by May and colleagues (1995). Each trial was initiated by an operator who monitored the activity of the cat on a video display. Each trial began with illumination of a green light-emitting-diode (LED) located at 0° azimuth. The green LED signaled the cat to depress a pedal to initiate a sequence of noise bursts, Rhythm 1, from a target source located at 0° azimuth. The target sequence was interleaved with a complementary sequence from a masker source that varied in location from trial to trial. After a variable hold time, the target rhythm changed from Rhythm 1 to Rhythm 2, and the cat was required to release the pedal to receive a food reward. The duration of the sequences of Rhythm 1 varied randomly from trial to trial with equal probability among 1200, 2400, 3600, or 4800 ms in what will be referred to as Hold 1, Hold 2, Hold 3, and Hold 4 trials, respectively. The Hold 4 trials were used only as catch trials for the Hold 3 condition.

Performance on each trial was scored according to the latency of pedal release relative to the time of the first sound burst that differed from Rhythm 1. That pattern change occurred 600 ms after the onset of Rhythm 2 and is indicated in Figure 1B by a vertical dashed line. Starting from that 600 ms time point, cats had a window of 1200 ms in which pedal releases were scored as “hits.” Releases more than 1200 ms prior to the beginning of the hit window were scored as “early release” and were not analyzed further. Releases 1200 to 0 ms prior to the beginning of the hit window were scored as “false alarms.” Releases after the 1200 ms hit window were scored as “misses.” The sounds ceased after the hit window, meaning that later times of pedal releases were of little interest and, therefore, were not recorded aside from counting them as misses. Hits were rewarded with delivery of a portion of pureed canned cat food (Purina Friskies). Early releases, false alarms, or misses triggered 4-s time-out periods, signaled by a flashing blue LED, in which there was no reward and in which no new trial could be initiated. Hold 2, Hold 3, and Hold 4 trials served as catch trials for Hold 1, Hold 2, and Hold 3 trials, respectively. For instance, a hit on a Hold 2 trial was also counted as a correct rejection (i.e., not a false alarm) for a Hold-1 trial. Pedal releases during the hit window during Hold 4 trials were rewarded but were not otherwise scored. The rationale for using Hold *N + 1* trials as catch trials for Hold *N* trials, rather than running separate catch trials, was that we obtained a roughly equal number of catch and non-catch trials for each hold time and we potentially could collect both a false-alarm datum and a hit or miss datum on each trial (except for the Hold 4 condition). Time windows in which responses were scored as hits and false alarms were equal in duration, meaning that random pedal releases had roughly equal probability of being scored as hits or false alarms.

Table 1 shows the number of trials that were scored for the broadband condition, i.e., excluding early releases, for each Hold time and each cat. The numbers of trials did not vary significantly across Holds 1, 2, and 3 (*χ*^2^_(2)_ = 0.087, *p* = 0.96, Friedman Test); the numbers of scored Hold 4 trials were somewhat lower, reflecting a higher probability of early releases in the lengthy time prior to the false-alarm time window in that condition.

**TABLE 1:** The number of trials for each Hold number that were included in the data analysis after exclusion of early releases

Cat	Number of scored trials			
	Hold 1	Hold 2	Hold 3	Hold 4
Mu	401	470	426	263
Bo	377	356	358	320
Go	322	268	345	238
Oz	256	340	210	57
Ma	179	378	247	152
St	306	301	301	148

Each column represents a Hold number and each row represents a particular cat

At the beginning of training for this task, the target sounds were presented without a masker, and Rhythm 2 was presented at a level 10 dB greater than that of Rhythm 1. In that phase, cats were rewarded for detecting the increase in sound level and/or the change in rhythm. The level difference between Rhythm 1 and 2 was decreased as the cat became more proficient at the task. When both rhythms were at the same level, the change from Rhythm 1 to 2 could be detected only on the basis of the temporal pattern. When a cat could detect the rhythm change reliably, a masker was introduced at the ±80° locations. As proficiency improved, the target and masker separations were gradually decreased. Once the animal was proficient with all hold times and varying masker locations, the hold times and masker locations were randomized from trial to trial. After training in the broadband-sound condition, training shifted to the high-band and then low-band conditions (3 cats) or low-band and then high-band conditions (3 cats). Once cats were proficient in all three pass-band conditions, the passband filter conditions were varied every 3–4 days. The reported performance includes a minimum of 10 testing blocks for each passband condition, where each block represents one day of training. Data from enough testing blocks were included for each passband condition for each cat to yield data from ≥20 trials for each target/masker separation.

Each training session lasted for as long as the animal was willing to work, typically around 30 min each day. The training periods varied from cat to cat, lasting several months to a year, followed by 3 to 11 months of data collection.

The psychophysical procedure used in the present study, hold-release, differed from the two-alternative forced-choice procedure used in our previous study of human listeners (Middlebrooks and Onsan 2012). Also, there were slight differences in the rhythms that were used. The rationales for those differences are considered in the Discussion.

### Data Analysis

Performance was measured by computing the discrimination index, *d’* (Green and Swets 1966) for each masker location:

$$ {d}^{\prime }=z\left({P}_{\mathrm{hit}}\right)\kern0.2em {\textstyle \hbox{-}}\kern0.2em z\left({P}_{\mathrm{false}\kern0.18em \mathrm{alarm}}\right) $$

For each masker location, the proportion of hits (*P*_hit_) was given by the number of hits divided by the number of hits and misses across Hold 1, 2, and 3 trials, and the proportion of false alarms (*P*_false alarm_) was given by the number of false alarms divided by the number of false alarms, hits, and misses across Hold 2, 3, and 4 trials. *P*_hit_ and *P*_false alarm_ were transformed to standard deviants (z-scores), and the difference in z-scores gave the discrimination index, *d’*. In some conditions, *P*_hit_ was 1 or *P*_false alarm_ was 0, meaning that the *z*-score was undefined. In those situations, the proportion of hits or false alarms on *N* trials was expressed as (*N −* ½)/*N* or (½)/*N*, respectively (Macmillan and Kaplan 1985). Values of *d’* for each cat and passband were plotted as a function of masker location. The masker location at which the interpolated plot crossed a criterion of *d’* = 1 or, in a separate computation, *d’* = 2 was used as the rhythmic masking release (RMR) threshold.

The distributions of thresholds were not normally distributed. For that reason, non-parametric statistics were used for comparison of median thresholds between conditions.

## **RESULTS**

We begin by characterizing observations that were specific to the cats’ performance of the hold-release behavioral task. Then, we compare performance among broadband, low-band, and high-band stimulus conditions that were intended to identify the acoustic cues that provide highest spatial acuity for cats.

### Task Performance

Cats performed the hold-release task enthusiastically, showing high hit rates for large target/masker separations, declining to chance performance for narrow separations. The positions of cats’ heads and pinnae were monitored on the video display. Cats learned early in training to direct their attention toward the green start light and the target sound source, both located at 0° azimuth. In the training trials in which maskers were first introduced, some cats made orienting movements of the head and pinnae toward masker sources at peripheral locations, but that behavior rapidly extinguished. During data collection, cats tended to keep their heads oriented toward the target source at 0° and their mobile pinnae in a fully forward position, seemingly focused on the target.

**FIG. 2:**
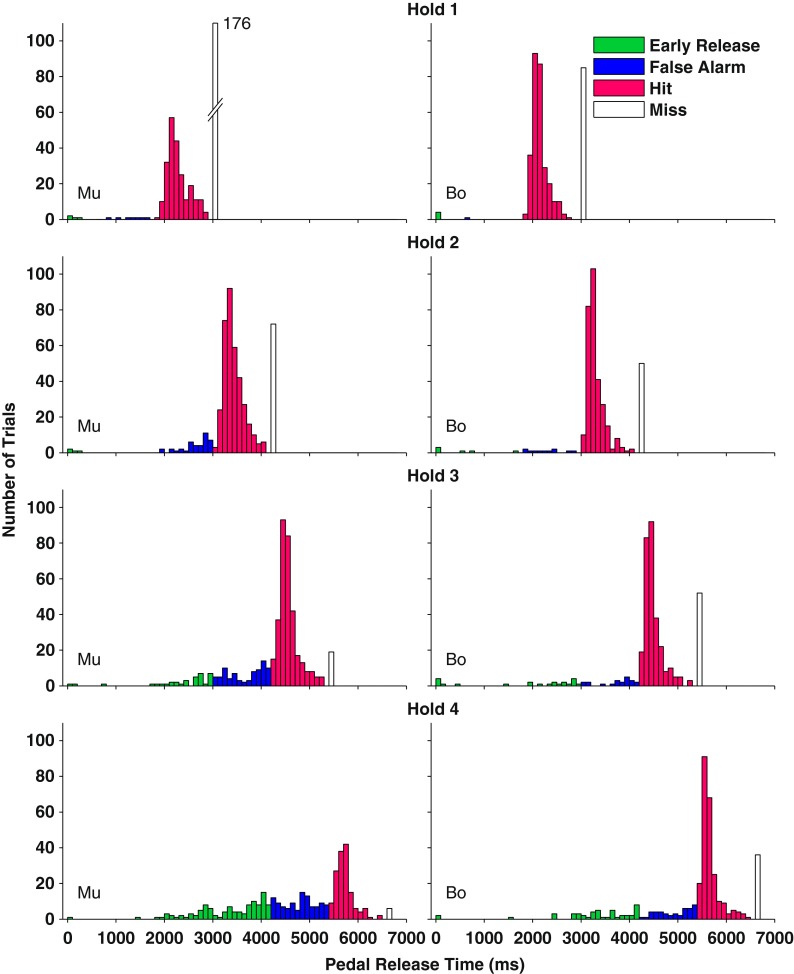
Latencies to pedal release for two cats in the broadband condition. *Each row of panels* represents a different Hold number (i.e., number of repetitions of Rhythm 1). *Colors on the bar graph* denote scores of early release (*green*), false alarm (*blue*), hit (*magenta*), and miss (*white*). *Columns of panels* represent individual cats (Mu and Bo). The 0 ms pedal release time denotes the start of the sound presentation.

The histograms in Figure 2 show the distributions of latencies to pedal release relative to onsets of the sound sequences; these data are from the broadband condition. Cats Mu and Bo are represented by the left and right columns of panels, respectively. The rows of panels represent the four hold times, i.e., the four durations of Rhythm 1 sequences prior to the change to Rhythm 2. Hold times, and the corresponding hit windows, were varied randomly from trial to trial in order to confound efforts to obtain reinforcement by releasing the pedal at some constant latency. In Figure 2, the bars are colored to represent responses that were scored as early releases (green), false alarms (blue), hits (magenta), or misses (white). Misses are grouped in single bars after the hit windows, regardless of how long the cat held the pedal after the offset of sound presentation. The histograms include results from all target/masker separations, including 0°, at which stream segregation was impossible, and ±5°, which proved to be narrower than the thresholds of any of the cats. The trials with those sub-threshold target/masker separations tended to increase the scatter of response latencies among early release, false alarm, and miss windows.

In each panel, the numbers of pedal releases were relatively low during presentation of Rhythm 1 (i.e., the hold time), and the numbers of responses increased sharply as the stimulus pattern changed to Rhythm 2, signaling the correct release time. Generally, the hit responses occurred with short latencies relative to the rhythm change, with 81 % of hits falling within the first half of the hit window across all cats, target/masker separations, and hold times. The observation that hit responses tended to fall early in the hit window indicates that the cats tended to respond as soon as they detected the increased inter-burst interval that characterized Rhythm 2; that is, the rhythms could be discriminated without listening to the entire rhythm. The numbers of early releases and false alarms increased with increasing hold time. Those increases were seen across all animals tested (*χ*^2^_(2)_ = 10.3, *p* = 0.0057, Friedman Test). We attribute the increase in early releases and false alarms with increasing hold times as indicative of the cats’ general impatience in waiting for reinforcement.

**FIG. 3:**
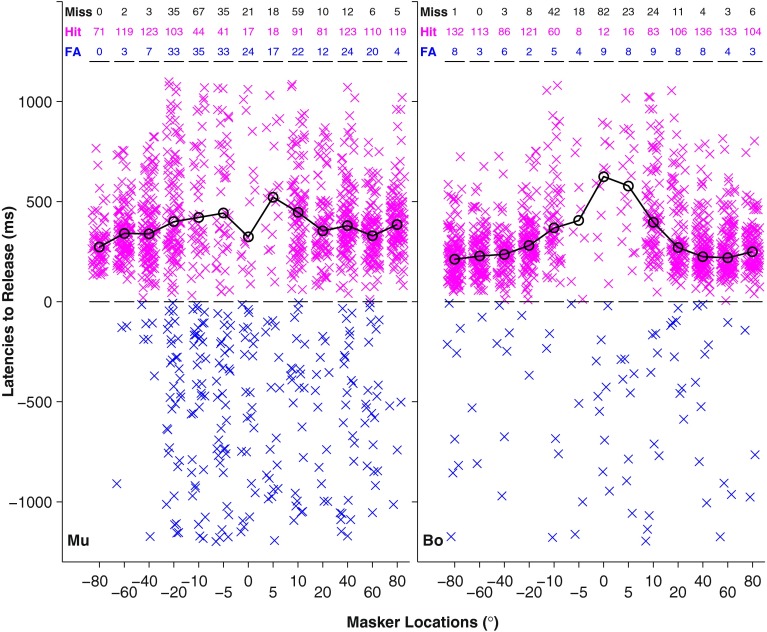
Latencies to release as a function of masker location for the same two animals shown in Figure 2 (Mu and Bo). Individual x symbols represent trials that were scored as hits (magenta) or false alarms (blue). The target was always located at 0°. Latency time point of 0 ms represents the start of the hit window. The time point of 1200 ms represents the end of the hit window. Trials below the 0 ms time point are false alarms. *Numbers at the top of the figures* denote the number of responses for misses, hits, and false alarms (FA) at that respective masker location. The *black curves* indicate the median latencies of hit responses.

Latencies to pedal release for hit responses as a function of masker location are shown in Figure 3, again in the broadband condition for cat Mu (left) and cat Bo (right). Symbols indicate the pedal release latency on each trial. Data are collapsed across Hold times 1, 2, and 3, and latencies are aligned relative to the time of the rhythm change (i.e., relative to the beginning of the hit window). Numbers of misses, hits, and false alarms are given by the rows of numbers at the top of the figure. At wide target/masker separations (e.g., Masker Locations ±80 and 60°), there were few false alarms, and there were many hits, typically early in the hit window. At narrower separation, the numbers of false alarms increased, the numbers of hits decreased, and pedal releases were later in the hit window. At near-zero separations, pedal releases were scattered fairly randomly throughout the false alarm and hit windows.

Cats exhibited a range of biases for or against releases of the response pedal. Cat Mu, whose data are shown on the left sides of Figures 2 and 3, was relatively eager to release the pedal. Compared to Cat Bo (on the right), Cat Mu had a higher false alarm rate at all but the widest target/masker separations. For the widest target/masker separations, shown in Figure 3, Cat Mu correctly rejected early pedal releases and released in the hit window. For narrow separations, however, he apparently was less able to segregate the target and masker sequences and, therefore, was less able to recognize Rhythm 1 during the Hold time. His tendency on such trials was to release early. The sum of his false alarms and hits consistently was higher than the number of his misses, and median latency of his hits in the 0° masker condition was relatively short. In contrast, Cat Bo shown on the right sides of Figures 2 and 3 was more conservative. His false alarm rates were low for all target/masker separations. His tendency on the difficult trials with separations ≤5° was to persist in holding the pedal, as indicated by low numbers of false alarms and hits, by large numbers of misses, and by the relatively long median latency for hits in the 0° masker condition. The *d’* analysis that was used for evaluating performance largely compensated for differences in response bias among cats. That is, values of *d’* in cases of bias toward pedal releases (like Cat Mu), which produced high numbers of false alarms but also high numbers of hits, could be roughly equal to *d’* values in cases of bias against release, which produced lower numbers both of false alarms and hits. The *d’* measures are presented in the next section.

### Broadband Spatial Stream Segregation

**FIG. 4:**
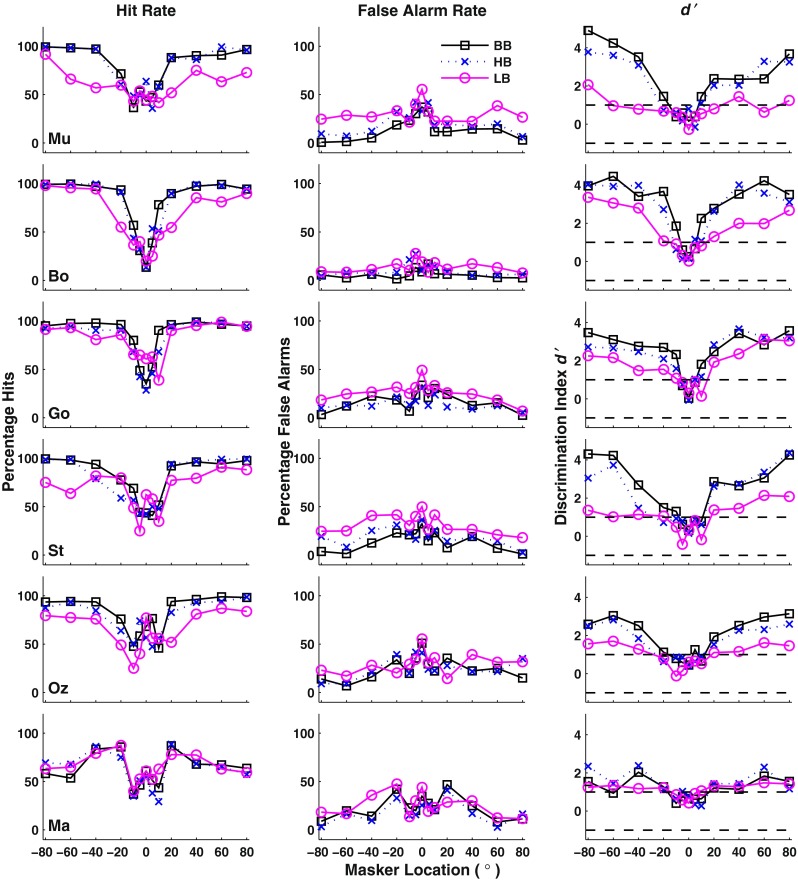
Task performance as a function of masker location. The three columns of panels show the proportion of hits, of false alarms, and the discrimination index for each filter condition. Filter conditions are denoted by *black solid lines* and *open squares* for broadband (BB), *blue dashed lines* and *x* symbols for high-band (HB), and *magenta solid lines* and *open circles* for low-band (LB). *Each row of panels* represents an individual cat.

Figure 4 shows the performance for all six cats, where each row of panels represents percentages of hits and false alarm rates and the *d’* for one animal. We first consider data from the broadband condition, indicated by open squares and solid black lines. Hit rates tended to be low at narrow target-masker separations (i.e., 0° and 5° masker locations), ≤50 % for most cats. Hit rates increased markedly with increasing target/masker separation, reaching 100 % for most cats. The dependence of false alarms on target/masker separation varied somewhat among cats. For the majority of cats (e.g., Cat Mu, top row), false-alarm rates were noticeably higher for narrow separations. For other cats (e.g., Cat Bo), false-alarm rates were largely insensitive to separations. The *d’* values (right column) were around 0 for near-zero target/masker separations and increased with increasing separations. In the broadband condition, most of the cats reached *d’* around 4, nearly 100 % correct, for the widest separations, although performance was not as good for Cats Oz and Ma. The two cats showing differing bias contrasted in the previous section—Mu the eager releaser and Bo the conservative—are represented in the top two rows of Figure 4. One can see that their *d’* values in the broadband condition were fairly similar even though Cat Mu’s hit and false-alarm rates both were noticeably higher than those of Cat Bo.

**FIG. 5:**
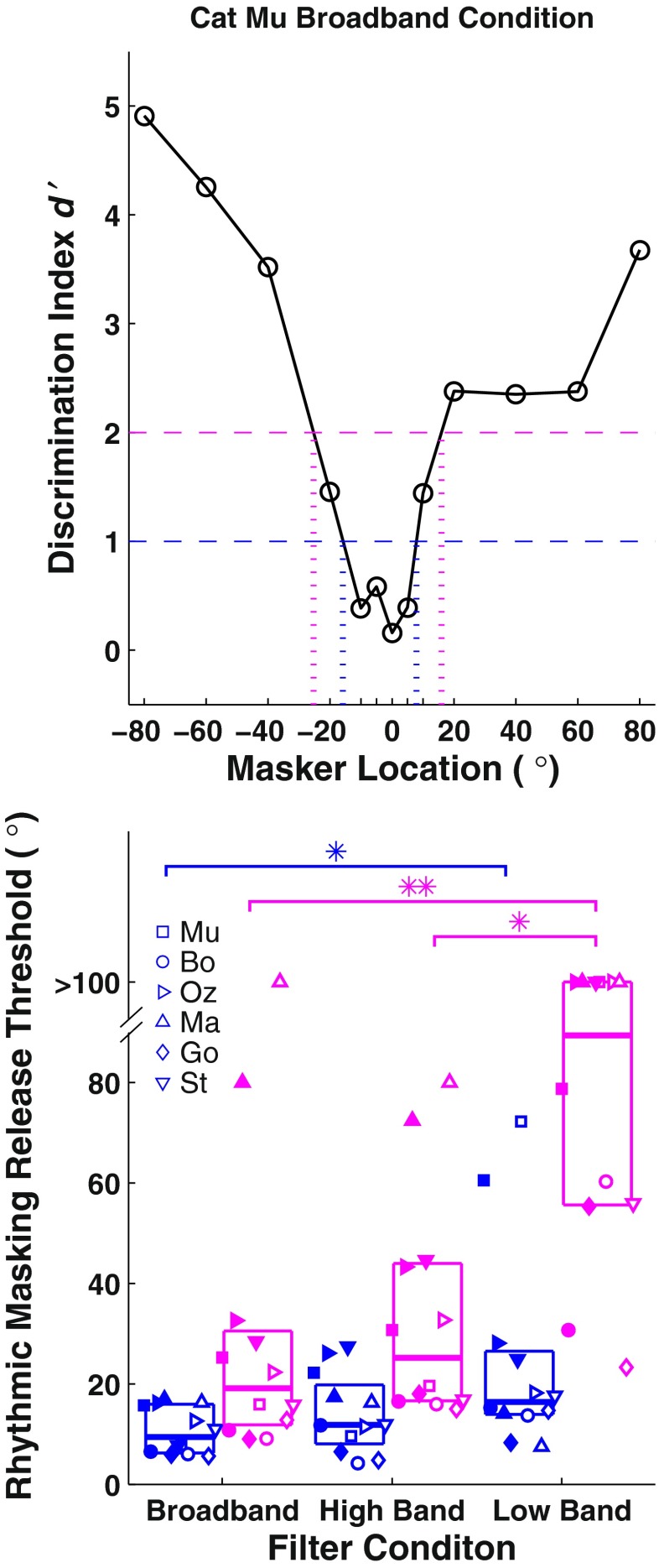
**A** Estimation of rhythmic masking release (RMR) thresholds. RMR thresholds were given by the masker locations to the left and right of the target at which the interpolated plot crossed a criterion of *d’* = 1 (*blue dashed line*) and, in a separate computation *d’* = 2 (*magenta dashed line*). **B** Distribution of RMR thresholds for each pass-band condition. Data shown in blue reflect the *d’* ≥ 1 criterion, and data shown in magenta reflect the *d’* ≥ 2 criterion. Each *symbol* represents a different cat. *Filled and open symbols* represent RMR thresholds located to the left and right of the target, respectively. A random horizontal offset is added to each symbol to minimize overlap between data points. The *horizontal lines* of each box represent the 25th, 50th, and 75th percentiles.

Rhythmic masking release (RMR) thresholds were given by the narrowest target/masker separation at which *d’* was consistently ≥1 and, in a separate computation, *d’* ≥2. The criterion of ≥1 (Fig. 5A, blue) was used to permit comparison with our previous study in humans (Middlebrooks and Onsan 2012), and the criterion of ≥2 (Fig. 5A, magenta) was used to better evaluate the difference in performance in the various passband conditions (presented below). Two RMR thresholds were recorded for each passband condition and *d’* criterion, for maskers to the left and the right of the target. The distributions of RMR thresholds in the broadband condition for all six cats are given in the left-most pair of columns in Figure 5B as box plots and with individual symbols. The median broadband RMR threshold was 9.4° for the *d’* ≥ 1 criterion and 19.1° for the *d’* ≥ 2 criterion.

### Performance in Conditions of Restricted Spatial Cues

We tested conditions of limited frequency bandwidth as a means of identifying the major acoustical cues that cats use for spatial stream segregation. The low-band condition used a spectrum limited from 0.4 to 1.6 kHz. We assume that essentially the only useful spatial cue in that frequency band is the ITD in temporal fine structure. The high-band condition used a spectrum of 4.0 to 25 kHz. In that band, the most likely cues involve differences in sound level, both in the form of interaural differences in levels (ILDs) and as differences in target and masker levels at each ear. There might also be some influence of ITDs in high-frequency sound envelopes, although Middlebrooks and Onsan (2012) demonstrated only a weak contribution of envelope ITD to spatial stream segregation by humans.

Spatial stream segregation in the high-band condition was nearly as high as that in the broadband condition. That can be seen for all cats in the *d’* plots on the right column of panels in Figure 4. The blue dotted lines indicating the high-band condition nearly overlie the black solid lines of the broadband condition. In contrast, performance was consistently degraded in the low-pass condition, in which low-frequency ITDs presumably are the spatial cue. In Figure 4, the magenta lines indicating the low-band condition generally show lower hit rates, higher false-alarm rates, and lower *d’*.

Distributions of RMR thresholds for the various passbands are shown in Figure 5B. Given the criterion of *d’ ≥* 1, median threshold values were 9.4° for broadband, 11.8° for high-band, and 16.4° for low-band. Median values varied significantly with passband (Friedman test, *χ*^2^_(2)_ = 7.8, *p* = 0.020). A post hoc analysis with Bonferonni adjustment showed that low-pass thresholds were significantly wider than broadband thresholds (*p <* 0.05*)* but that there was no significant difference between broadband and high-band or between high-band and low-band thresholds (*p >* 0.05). The dependence of performance on stimulus passband was greater given a criterion of *d’ ≥* 2. Median threshold values were 19.1° for broadband, 25.2° for high-band, and 89.4° for low-band. Median values varied significantly with passband (Friedman test, *χ*^2^_(2)_ = 19.3, *p* < 0.0001). The post hoc analysis with Bonferonni adjustment showed that low-band thresholds were significantly wider than broadband thresholds (*p <* 0.01*)* and wider than high-band thresholds (*p* < 0.05) but, again, that broadband and high-band thresholds were not significantly different (*p* > 0.05).

**FIG. 6:**
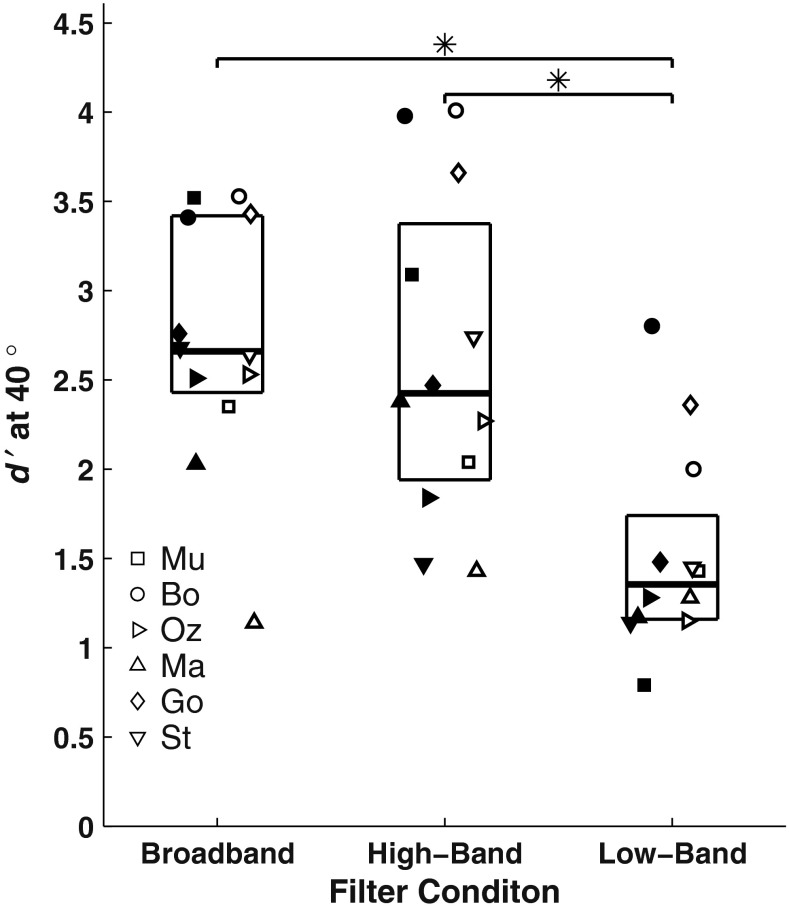
Distribution of d’ values for 40° target/masker separations for each pass-band condition. Each *symbol* represents a different cat. *Filled and open symbols* represent *d’* for maskers located to the left and right of the target, respectively. A random horizontal offset is added to each symbol to minimize overlap between data points. The *horizontal lines* of each box represent the 25th, 50th, and 75th percentiles.

We also compared across passbands the distributions of *d’* for target/masker separations of 40° (Fig. 6). The 40° separation was chosen because that separation tended to produce *d’* higher than the threshold value of *d’* = 1 and lower than asymptotic values for nearly all cats and conditions. Median values of *d’* at the 40° separation were 2.66 for broadband, 2.42 for high-band, and 1.36 for low-band. The *d’* values varied significantly with passband (Friedman test, *χ*^2^_(2)_ = 17.3, *p* = 1.7 × 10^−4^). A post hoc analysis with Bonferroni correction showed that *d’* values were significantly higher (i.e., performance was better) for broadband and high-band than for low-band conditions (*p* < 0.05) but that there was no significant difference between broadband and high-band conditions (*p* > 0.05).

Overall, the results showed little or no impairment of spatial stream segregation by cats when low-frequency ITDs were made unavailable and a severe degradation in performance when high-frequency cues were eliminated. These results make an interesting contrast to the situation in humans, in which performance is substantially better with low-band than with high-band sounds (Middlebrooks and Onsan 2012).

## **DISCUSSION**

Cats performed this RMR task reliably, exhibiting spatial stream segregation comparable to that of humans. The median RMR threshold in the broadband condition for 6 cat listeners was 9.4°, only slightly broader than the corresponding median of 8.1° for 7 human listeners (Middlebrooks and Onsan 2012). Cats differed from humans in that performance by the cats was better in the high-band than the low-band condition, whereas the opposite was true for humans. Possible reasons for that difference are considered in a later section.

The hold-release task used in the present study differed from the task used in the previous human psychophysical study (Middlebrooks and Onsan 2012). The human study used a one-interval, two-alternative design: Rhythm 1 or Rhythm 2 was presented on each trial with equal probability, and the listener responded by pressing one of two keys. Our initial efforts to train cats on the two-alternative task were unsuccessful, largely because the cats tended to associate one or the other response pedal with the location of the masker source rather than with the stimulus rhythm. The cat and human studies also differed somewhat in that the rhythms in the present cat study were extended from 800 to 1200 ms by adding two bursts to target and masker patterns. The reason for that change was to provide the cats with a longer time window in which to release the pedal during the Rhythm 2 presentation. Informal comparisons by human listeners produced essentially equal RMR thresholds between the two-alternative and hold-release tasks. Finally, the upper limit of the broadband and high-band conditions was extended to 25 kHz to take advantage of the cat’s higher audibility range.

### Species Differences in Use of Spatial Cues

In the present study and the previous human psychophysical study (Middlebrooks and Onsan 2012), stimulus bandwidths were manipulated to limit the available spatial cues. The low-band condition was intended to minimize use of cues related to sound levels, and the high-band condition was intended to eliminate usable cues from ITDs in temporal fine structure. Cats consistently performed worse in the low-band than in the high-band and broadband conditions in that hit rates were lower, false-alarm rates were higher, *d’* values were lower, and RMR thresholds were broader in the low-band condition. High-band performance, in contrast, was not significantly different from that in the broadband condition. We take this to mean that in the broadband condition, cats relied primarily on high-frequency ILD cues. This contrasts with the previously reported human results in which RMR thresholds in low-band and broadband conditions were not significantly different and thresholds in the high-band condition were significantly broader. Those results suggest that in the broadband condition, humans relied primarily on ITD cues.

The superior performance by cats in the high-band condition agrees with expectations based on single-unit recordings from cortical area A1 in anesthetized cats (Middlebrooks and Bremen 2013). In that study, cortical neurons demonstrated a correlate of spatial stream segregation by synchronizing preferentially to one of two interleaved sequences of broadband noise bursts from sound sources that were separated in location. Neurons that were most sensitive to frequencies greater than 4 kHz tended to show higher *d’* for segregation of sound sequences from alternating sources than did neurons that were most sensitive to lower frequencies. A test of the ability of a linear classifier to discriminate stimulus rhythms based on neural spike patterns also showed good performance among the highest-frequency neurons although, inexplicably, that test also showed good performance among the small sample of units that were most sensitive to frequencies around 500 Hz.

The poorer performance by cats in the low-band condition conflicts with early studies of localization of pure tone stimuli. Casseday and Neff (1973) trained cats to walk to one of two possible pure-tone sources located symmetrically about the frontal midline, and Martin and Webster (1987) used a conditioned-avoidance task in which cats were required to detect a change in the location of a tone source away from the frontal midline. In both of those studies, performance was best for tone frequencies ≤2 kHz, poor at 4 kHz, and improving (Casseday and Neff 1973) or irregular (Martin and Webster 1987) at even higher frequencies. The reason for the difference in frequency dependence between previous and present studies is not obvious, but we note that the stimulus conditions were very different. In the early localization studies, tone bursts were 500 ms in duration and were repeated five or more times for each location judgment. Those lengthy sound presentations would have permitted a cat to move its head and ears relative to the sound source during individual sound bursts and thereby obtain dynamic localization cues. In the present study, in contrast, individual sound bursts were only 20 ms in duration. The sequences of such bursts lasted for some seconds, but the task required the cats to segregate successive 20-ms bursts from the two sources in order to evaluate the rhythm conveyed by the sequence from one or the other source. We also note that, in humans, discrimination of the locations of two successive sounds (i.e., a minimum audible angle test) was not a good predictor of the effects of passband on RMR thresholds (Middlebrooks and Onsan 2012).

Cats in the present study made less effective use of low-frequency spatial cues than do humans (Middlebrooks and Onsan 2012). That inter-species difference likely can be attributed primarily to differences in the sizes of cat and human heads, resulting in differences in interaural delays. In both species, ITDs vary somewhat with frequency across the 0.4-to-1.6 kHz range of our low-band stimulus. In cats, ITDs at 0.8 kHz are around 100 and 320 μs for sound sources at 15 and 90°, respectively (Roth et al. 1980). In humans, ITDs are ∼1.5-to-2 times greater for the same source angles: 140 and 660 μs, respectively, on a human-sized mannequin (Kuhn 1977). Despite the differences in the ranges of ITDs that cats and humans typically experience, their sensitivity to ITD is similar. Reported just-noticeable differences in ITDs are around 25 μs in cats (Wakeford and Robinson 1974; Cranford 1979) and between 9 and 45 μs in human depending on listeners’ degree of training (Zwislocki and Feldman 1956; Klumpp and Eady 1956; Wright and Fitzgerald 2001; Middlebrooks et al. 2013). Also, the just-noticeable difference for ITD increases dramatically or becomes immeasurable at tone frequencies greater than ∼1.5 kHz in both cats (Wakeford and Robinson 1974) and humans (Zwislocki and Feldman 1956; Klumpp and Eady 1956; Brughera et al. 2013). The cat’s smaller head means that, given comparable ITD sensitivity in cats and humans, the displacement of a sound source from the midline needed to achieve a just-noticeable ITD is 1.5-to-2 times larger for a cat than for a human. Scaling of ITDs by a factor of 1.5 to 2 would reduce to some degree the difference in median low-band RMR thresholds between cat (16.4°) and human (5.9°). One can see in Figure 4, however, that a simple scaling of ITD would not bring the psychometric functions for the low-band condition in line with those for broadband and high-band conditions. That is, cats’ maximum *d’* levels of performance in the low-band condition rarely reached the high levels of performance attained in the broadband or high-band condition. We conclude that the cat’s smaller head size relative to humans almost certainly contributes to the cat’s less effective use of low-frequency cues, but that head size cannot entirely account for the inter-species difference.

We considered two other factors that might explain to some degree the cats’ relatively poor performance in the low-band condition. One consideration is that detection thresholds by cats are reported to be as much as ∼15 dB higher for sounds in the low-frequency compared to the high-frequency bands that were tested (Neff and Hind 1955; Heffner and Heffner 1985). We note, however, that our low-band stimuli were ∼50 dB above the reported audiograms for cats and, therefore, should have been clearly audible. Also, in pilot studies, we observed that 5-dB increases in the levels of low-band sounds failed to improve performance. The second consideration is simply the observation that cats were less willing to perform the task in the low-band condition. That might be because, for one reason or another, their performance was worse in that condition so that they received less frequent reinforcement. Alternatively, it might have been that the low-band stimulus was, for some reason, aversive to the cats.

Cats in the present study performed better in the high-band condition (median threshold 11.8°) than they did in the low-band condition (16.4°) and better than humans in the high-band condition (median threshold 15.4°; Middlebrooks and Onsan 2012). We assume that the principal cues for spatial stream segregation in the high-band condition are related to sound level rather than ITD (Middlebrooks and Green 1991; Macpherson and Middlebrooks 2002; Middlebrooks and Onsan 2012). Other things being equal, we would expect that, at any particular frequency, the cat’s smaller head would produce weaker refraction and weaker level-related cues than would the larger human head. Two factors mitigate the possible disadvantage of a smaller head. First, the cat’s audible range extends more than an octave higher than that of humans. In the human study, the high-band stimulus cut off at 16 kHz, which is well above the most sensitive frequency region of listeners. In the cat study, however, the high-band stimulus extended to 25 kHz, which is within the sensitive portion of the cat’s behavioral audiogram (Heffner and Heffner 1985). The higher-frequency hearing by the cat would permit it to benefit from the decrease in wavelengths at higher frequencies, which would result in stronger level cues (Middlebrooks and Pettigrew 1981; Phillips et al. 1982; Tollin and Koka, 1990a). Second, the directional sensitivity of the cat’s external ears could have enhanced spatial level cues around the frontal midline. Previous acoustical measurements have shown that when the cat’s ears are oriented frontally, as they were during task performance, the axes of greatest sensitivity are located ∼10–40° from the frontal midline, meaning that sensitivity tends to decline fairly steeply across the midline (Middlebrooks and Pettigrew 1981; Calford and Pettigrew 1984; Middlebrooks and Knudson 1987; Musicant et al. 1990; Young et al. 1996; Tollin and Koka 2009a). The resulting interaural level differences show a particularly steep gradient across the midline in cat at frequencies above ∼8 kHz (Middlebrooks and Pettigrew 1981; Musicant et al. 1990; Tollin and Koka 2009b). In contrast, the human ear at the highest audible frequencies is focused near the frontal midline, meaning that the spatial gradient of levels in the central ∼ ±15° is relatively flat (Middlebrooks et al. 1989).

There are three mechanisms by which the spatial dependence of sound levels at the ears could support spatial stream segregation. The *first* would be a conventional use of ILDs as spatial cues, resulting in differential representations of locations of target and masker sources. The *second* would be a “better ear” mechanism in which the cat could attend to the ear contralateral to the masker source, thereby optimizing the target-to-masker ratio. The *third* mechanism would be detection of differences in the levels of target and masker sounds at each ear, exploiting the potentially audible rhythms of varying sound levels. Humans can segregate two interleaved sequences of sounds that differ in level by as little as 3 dB, even when the two sources are co-located (Middlebrooks and Onsan 2012). The human study showed that that target/masker level cues were weaker than the cue given by ILDs. In cats, however, the steeper gradient of high-frequency sound levels around the midline might enhance the contribution of absolute-level cues and, thereby, account for the cat’s superior performance in the high-band condition.
